# Improving Enamel Acid Resistance by an Intraoral Fluoride-Release Device Incorporating Cationic Hydroxy Cellulose Gel Using 3D Printer Molding

**DOI:** 10.3390/ma17235731

**Published:** 2024-11-23

**Authors:** Ryouichi Satou, Kento Odaka, Ryo Sako, Masatoshi Ando, Naoki Sugihara

**Affiliations:** 1 Department of Epidemiology and Public Health, Tokyo Dental College, Tokyo 101-0061, Japan; sugihara@tdc.ac.jp; 2 Department of Biomedical and Applied Sciences, Indiana University School of Dentistry, Indianapolis, IN 46202, USA; mando@iu.edu; 3 Department of Oral and Maxillofacial Radiology, Tokyo Dental College, Tokyo 101-0061, Japan; odakakento@tdc.ac.jp; 4 Department of Endodontics, Tokyo Dental College, Tokyo 101-0061, Japan; sakoryou@tdc.ac.jp

**Keywords:** fluoride, 3D printer, slow-release, intraoral fluoride-releasing device, demineralization, enamel

## Abstract

An intraoral fluoride-releasing device (IFRD) is a cost-effective tool for introducing fluoride into the oral cavity. It allows prolonged uptake of low concentrations of fluoride into teeth. We developed a new IFRD using 3D additive manufacturing and a new low-release fluoride gel. Gels for IFRDs were synthesized from hydroxyethyl cellulose (SE600) and cationic hydroxyethyl cellulose (L200). We compared the effects of the new cationic fluoride slow-release gel and non-cationic gel on enamel acid resistance in vitro. Cationization significantly increased fluoride ion concentration, as evident from its concentrations of 0.68 ± 0.08 ppm for SE600 and 4.24 ± 0.83 ppm for L200 after 60 min of immersion in distilled water. In addition, the acid resistance of bovine tooth enamel post-application was analyzed by measuring tooth loss, mineral loss (ΔZ), and lesion depth (Ld) using polarized light microscopy, electron microscopy, and micro-radiography. Compared to the SE600 group, the cationic L200 group had significantly reduced ΔZ and Ld, enhancing enamel acid resistance. This device could be implemented in areas where adequate oral care is challenging, including preventive dentistry, ward management, nursing homes, and dental clinic visits.

## 1. Introduction

The caries-preventive actions of fluoride are multifaceted and primarily strengthen the tooth structure and inhibit cariogenic bacterial metabolism [1,2,3]. For instance, it enhances enamel acid resistance, aids remineralization, and prevents demineralization, which is influenced by its presence in the saliva [1,4,5]. Fluoride ions (F^−^) form calcium fluoride on tooth surfaces in high concentrations, which is eventually converted to less-soluble fluorapatite [1,3,4,5,6]. Clinical applications of fluoride include topical and systemic use [1,3]. Epidemiological and laboratory studies have demonstrated that the topical application of fluoride is more effective than its other forms [1,2,3,6,7,8]. Recently, dental materials that release small amounts of F^−^ over extended periods have been developed, such as fluoride-releasing aluminosilicate compomers and glass-ionomer cement used as sealants in children [9,10,11,12]. However, these materials provide only secondary effects because of their slow release, making it difficult to maintain a constant oral fluoride concentration [10,13,14,15,16]. Moreover, a trade-off exists between F^−^ release and the mechanical properties of restorative materials; materials with high fluoride release often have low compressive strength, limiting their applications in load-bearing areas [17,18]. Therefore, new materials and preventive methods focusing primarily on releasing F^−^ and maintaining salivary fluoride concentrations need to be developed [12,19].

The intraoral fluoride-releasing device (IFRD), developed by the Southern Research Laboratory in the U.S. and UK, maintains a consistent fluoride concentration in the oral cavity over extended periods [10,12,19]. It is a cost-effective device for incorporating low fluoride concentrations into tooth structures [10,12,19,20,21]. An IFRD serves as an intermediary between topical and systemic applications [10,12], increasing the levels of fluoride in saliva and dental plaques without significantly affecting serum fluoride levels [12,13,19,22]. Researchers believe it can enhance fluoride concentration in the oral environment, improve tooth tissue acid resistance, and promote remineralization of early carious lesions [10,12]. However, it is not yet clinically approved due to its developmental stage and lack of clinical trials [12,13,19,20,21]. IFRDs could be beneficial for high-caries-risk populations and those with irregular dental visits, partial dentures, hyposalivation, and those undergoing orthodontic treatment [12,19].

Because F^−^ is negatively charged, efforts have been made to enhance its adsorption in toothpaste by cationization (adding a positive charge to the base material) [23,24]. Hydroxyethyl cellulose (HEC), with a CAS number of 9004-62-0 and a molecular formula of C_29_H_52_O_21_, is a highly water-soluble polymer [25]. HEC forms a viscous, clear solution in water and serves as a thickener, suspending agent, and protective colloid [25]. It has been used as a pharmaceutical additive in dental and oral medications [25], with over 40 years of use in cosmetics, catalysts, paints, and construction materials [25]. Human studies have proved it to be safe with no skin or eye irritation or allergic reactions, thus confirming its safety for cosmetic formulations and regular use [23,24,25]. Cationized HEC is produced by adding glycidyl trimethyl ammonium chloride to HEC in the presence of an alkali, which increases its hydrophilicity and viscosity [25]. This cationic HEC is extensively used in cosmetics and fragrances, particularly in shampoos, and has been used as a toothpaste ingredient in both dentistry and medicine [23,24,25].

We aimed to develop a new gel for IFRDs using cationic HEC for sustained fluoride release and prolonged fluoride concentration in the mouth. In addition, we attempted to construct a new palate-mounted IFRD customized for patients using optical impressions and 3D printing. This device releases low-concentration fluoride over an extended period, allowing patients to control the F^−^ release and duration by adjusting the amount and composition of the gel. Additive manufacturing with biocompatible materials enables the rapid and simultaneous production of devices for multiple patients. Complex hollow structures and edge designs are now easily achievable, enhancing design flexibility [26,27,28]. In the present study, we developed a new fluoride-releasing device using additive manufacturing technology for IFRDs and demonstrated its caries-inhibitory effects in vitro in the form of its ability to enhance enamel acid resistance.

## 2. Materials and Methods

### 2.1. Preparation of Enamel Samples

We used 48 bovine mandibular incisors. The enamel samples (dimensions: 5 (W) × 5 (H) × 5 (D) mm) were accurately fashioned and thoroughly polished using water-resistant abrasive papers (grit sizes: #1000, #2000, and #4000) to obtain a mirror-like surface finish.

### 2.2. Preparation of Cationic Fluoride Slow-Release Gel for IFRD

A non-cationized normal HEC (SE600; Daicel Miraizu Ltd., Tokyo, Japan) and cationic HEC (CELQUAT L-200; Polyquaternium-4, Nouryon, Amsterdam, The Netherlands) were used to produce the IFRD gel according to a previously described method in the Japan Patent Office’s published patent JPA2008007475 and the method for producing cationized cellulose [23,24]. The composition of the gel was identified in the patents as “hydroxyethylcellulose dimethyldiallylammonium chloride”, a type of cellulose. HEC is water-soluble, stable in the neutral range of pH 6.5–8.0 (20 g/L, 25 °C), and is tasteless and odorless. The HEC powder was dissolved in distilled water to prepare a 1% (*w*/*w*) aqueous solution, to which 1.4% (*w*/*w*) NaF (CAS 7681-49-4; FUJIFILM Wako Pure Chemical Corp., Tokyo, Japan) was added and the solution was stirred for approximately 24 h. Afterward, the solution was dialyzed for 24 h using an artificial dialysis membrane (Spectrum Spectra/Por™ 6 Pre-wetted Standard RC Dialysis Tubing; 1–50 kD MWCO, Thermo Fisher Scientific Inc., Waltham, MA, USA) to remove unbound free ions. The dialyzed solution was concentrated using a rotary evaporator (Rotavapor^®^ R-100; BUCHI Corp., New Castle, DE, USA), diaphragm-type dry vacuum pump (DAP-15; ULVAC KIKO Inc., Miyazaki, Japan), and recirculating chiller (F-100 recirculating chiller; BUCHI Corp., New Castle, DE, USA) until a non-flowable gel was obtained (Supplementary Figure S1).

### 2.3. Measurement of Fluoride-Release Ability of Gels Using Ion Electrode Method

The release of F^−^ from the gel was measured by placing the gel in distilled water and determining the free F^−^ concentration using the ion electrode method. A 10 mL solution was prepared by combining 9 mL of Milli-Q water and 1 mL of TISABIII (TISAB III solution; 89467, Sigma-Aldrich, Burlington, MA, USA) in a 15 mL plastic vial (Push Vial 15 mL; PV-15, Nikko Company, Ishikawa, Japan). A 200 mg sample gel was added to the vial. The solution was maintained at 37 °C and the measurements were obtained with stirring at 50 rpm. An F^−^ electrode (6561S-10C, HORIBA, Ltd., Kyoto, Japan), F^−^ electrode tip (7661S, HORIBA, Ltd., Kyoto, Japan), and ion meter (LAQUA F-73, HORIBA, Ltd., Kyoto, Japan) were used. Eight samples from each SE600 gel and L200 gel group were measured continuously for 3 h at 10 s intervals.

### 2.4. Design of an Artificial Saliva Reflux Device for IFRD Gel

**Figure 1:**
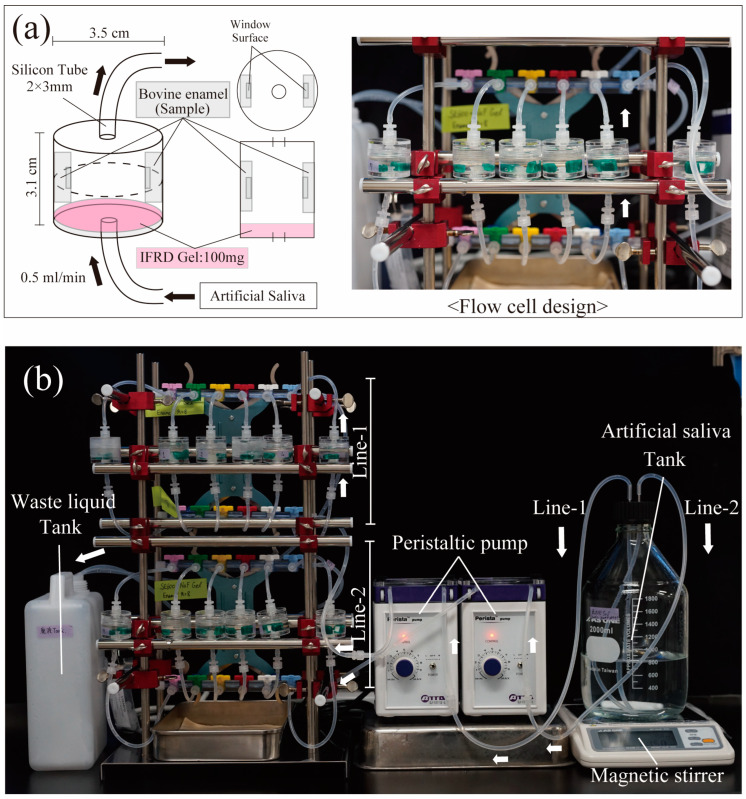
Design of the artificial saliva reflux device for IFRD gel. (**a**) Overview of the flow cell for the artificial saliva reflux device, including the design drawing (left) and operational flow cell (right). Arrows indicate the direction of the flow of the solution. (**b**) The solution was pumped from the artificial saliva tank by a peristaltic pump, returned to each cell of Line-1 (top) and Line-2 (bottom), and subsequently disposed to the waste tank. Each line could handle up to 12 samples simultaneously, allowing the entire device to process 24 samples simultaneously.

We designed an IFRD to simulate the oral environment. A schematic of the device is depicted in Figure 1. It consisted of a flow cell containing two bovine enamel samples and 100 mg of the gel (Figure 1a). The cell was filled with artificial saliva, and the samples were glued to the wall. The sample window surface was exposed toward the center of the cell. Holes were drilled into a small acrylic case (φ3.5 × 3.1 cm, 7732900, Ryohin Keikaku Co., Ltd., Tokyo, Japan) to attach the silicone tubes (Laboran Silicone Tube 2 mm × 3 mm, 9-869-04, AS ONE Corp., Tokyo, Japan). The cell was replaceable and could easily be opened and closed using a central screw (dotted line in Figure 1a). Artificial saliva, homogenized by stirring, was circulated from a tank to a flow cell and subsequently to a waste tank (Figure 1b) at a rate of 0.5 mL/min. The solutions were freshly prepared to prevent their deterioration during storage. This device employed a peristaltic pump and was similar to those used by Cate et al. and Lippert et al. [29,30], with modifications for larger, detachable cells.

### 2.5. Enamel Acid Resistance Experiment (pH-Cycling)

We equally divided the samples into three groups: (1) control (*n* = 8, untreated fluoride, 0 ppm F, pH 7.0), (2) SE600 (*n* = 8, non-cationic hydroxyethyl cellulose, HEC DAICEL SE600-based NaF-saturated gel), and (3) L200 (*n* = 8, cationic hydroxyethyl cellulose, CELQUAT L-200-based NaF-saturated gel). Half of the mirror-polished enamel surfaces were coated with dental wax to obtain experimental and control surfaces on the same enamel (Inlay Wax Soft; 27B2X00008000028; GC Co., Ltd., Tokyo, Japan).

We used a pH cycling method with two phases repeated four times: the IFRD gel treatment phase, which simulated oral conditions during gel use, and the demineralization phase, which used a lactate buffer solution. The IFRD gel treatment phase was set to 8 h, reflecting the average sleep time. An artificial saliva reflux device was used for the IFRD gel for each phase. The demineralization solution consisted of 0.2 M lactic acid buffer, 1.5 mM Ca, 0.9 mM P, pH 4.5, 5.5 DS for 6 h at 37 °C, and the artificial saliva consisted of 0.02 M HEPES-based buffer, 1.5 mM Ca, 0.9 mM P, pH 7.3, 5.5 DS for 8 h at 37 °C.

### 2.6. Three-Dimensional Laser Microscopy

After the wax was removed, the samples were dehydrated using an ethanol series (ascending). The differences in step height profiles between the experimental surface (ES) and reference surface (RS) following pH cycling were studied using a three-dimensional (3D) laser microscope (LEXT OLS4000; Olympus Corp., Tokyo, Japan). Images were acquired at the boundary between the acid-demineralized ES and wax-protected RS to evaluate tooth defects arising from an acid challenge within a 645 µm × 645 µm area. In addition, the average roughness (Sa) of the ES in a 645 µm × 645 µm area, with a cut-off value of 80 µm, was determined. The number of substantial defects and Sa were measured at five distinct points per sample at the boundary between ES and RS. The mean and standard deviation (SD) values were calculated.

### 2.7. Reaction Area Depth Measurement Using a Polarizing Microscope

The method of Ogata et al. was used to prepare and analyze the enamel sections using polarizing microscopy [31]. The resin-embedded samples for SEM observation were sliced into 120 μm thick sections, which were ground into 100 μm sections using an internal annulus saw microtome (Leica 1600, Leica Microsystems, Heidelberg, Germany). Images were obtained using a polarizing microscope (ECLIPSE E600 Pol, Nikon Corp., Tokyo, Japan) and a camera (Axiocam ERc 5s, Carl Zeiss Co., Ltd., Jena, Germany). The reaction area depth (RAd, μm) was measured using image analysis software (ver 3.3, ZEN lite, Carl Zeiss Co., Ltd., Jena, Germany). RAd was defined as the depth from the enamel surface to the deepest line showing discoloration due to changes in the refractive index from demineralization. It was measured at five locations per sample, and the mean SD values were calculated.

### 2.8. Cross-Sectional Morphology Assessment Using Scanning Electron Microscopy

After pH cycling, each sample was rinsed with xylene. The surface of analyte samples was treated using the carbon vapor deposition technique. Tooth surfaces were examined using a scanning electron microscope (SU6600; HITACHI Ltd., Tokyo, Japan) at an accelerating voltage of 15 kV.

### 2.9. Contact Microradiography

Imaging conditions and analysis methodology for contact microradiography were derived from Sato et al., referencing Angmar’s formula [32,33]. Samples were embedded in a polyester resin (Rigolac; Nisshin EM, Tokyo, Japan) to prepare 100 µm thick polished sections. We employed soft X-ray imaging that utilized a 20 µm Ni filter, whereas light microscopy at 200× magnification employed a glass plate (high-precision plate, HRP-SN-2; Konica Minolta, Tokyo, Japan). The following parameters were used for imaging with the CMR-3 system (Softex, Tokyo, Japan): tube voltage, 15 kV; tube current, 3 mA; and radiation time, 8 min. Images were analyzed using the Image Pro Plus software (version 6.2; Media Cybernetics Inc., Silver Spring, MD, USA) and an image analysis system (HC-2500/OL; OLYMPUS Corp., Tokyo, Japan) to obtain concentration profiles. Mineral loss (ΔZ) and lesion depth (Ld) were measured to compare demineralization. The values were converted to a histogram, where 0% represented no mineral and 100% corresponded to healthy enamel. Ld was defined as the distance from the enamel surface to the lesion where the mineral content exceeded 95% of that of sound enamel.

### 2.10. Fabrication of the Prototype Human Maxillary IFRD Device Using a 3D Printer

We designed a prototype maxillary IFRD device using CAD software (ver 18.0 3-matic medical; Materialize, Leuven, Belgium) on a maxillary dental model (D18FE-500E; Nissin Dental Products Inc., Kyoto, Japan). The shape of the maxillary dental model was captured as an STL file using an optical scanner (Atos Core; GOM, Braunschweig, Germany). Initially, an outline with a thickness of 5.0 mm was delineated to fit the lingual aspects of the dentition and the palatal region, followed by a surface clearance of 0.2 mm and removal of undercuts. Next, a 15 mm long × 3 mm wide × 0.5 mm deep cylinder was designed for the IFRD gel storage; 0.5 mm diameter cylinders were connected to each tooth surface. The device was divided into upper and lower sections to traverse the tank, and notches were added for repositioning and connection. The IFRD device was produced using a 3D printer (Objet 260 Connex; Stratasys Ltd., Eden Prairie, MN, USA). A biocompatible transparent material (MED 610, Stratasys Ltd.) was used for the device surface and tank, and a white material (FullCure835 VeroWhitePlus, Stratasys Ltd.) was used for the notch.

### 2.11. Statistical Analysis

The mean ± SD of eight samples was determined for each sample group. The release of F^−^ for the SE600 and L200 groups was compared using a non-paired *t*-test, with significance set at *p* < 0.05. Statistical significance for experiments involving three groups was assessed using one-way analysis of variance (ANOVA) (*p* < 0.05), followed by Bonferroni’s post hoc tests. Data were analyzed and graphs were generated using the Origin 2023 software (ver. 10.0.0.154; Lightstone Corp., Tokyo, Japan).

## 3. Results

### 3.1. Amount of F^−^ Released from SE600 and L200 Gels

**Figure 2:**
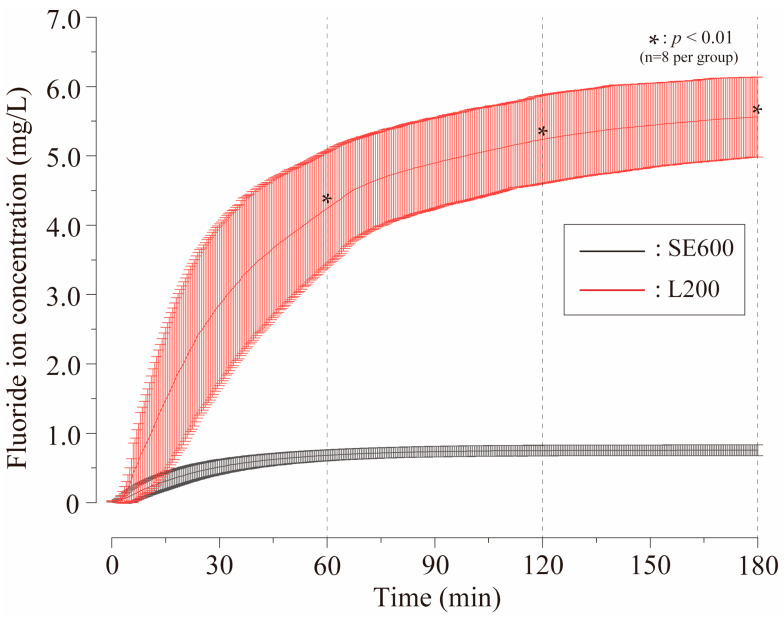
Changes in F^−^ intake over time in the SE600 and L200 groups. The graph depicts the mean ± SD of F^−^ concentration in the non-cationized SE600 and cationized L200 groups, which was measured continuously for 3 h at 10 s intervals (*n* = 8/group). Significant differences in the concentrations were noted between the two groups at 60, 120, and 180 min (* *p* < 0.01).

Figure 2 shows the concentration of F^−^ in the SE600 and cationized L200 groups over 3 h (mean ± SD, *n* = 8). The SE600 group demonstrated a gradual increase until 60 min, and subsequently stabilized (0.682 ± 0.079 mg/L at 60 min, 0.749 ± 0.077 mg/L at 120 min, and 0.756 ± 0.079 mg/L at 180 min). The concentration of F^−^ in the L200 group initially increased substantially, followed by a continued slow rise (4.241 ± 0.825 mg/L at 60 min, 5.240 ± 0.635 mg/L at 120 min, and 5.560 ± 0.574 mg/L at 180 min), with 5 to 8 times higher F^−^ release than that in the SE600 group (*p* < 0.01).

### 3.2. Three-Dimensional Laser Microscopy Measurements of Surface Profiles After pH Cycling

**Figure 3:**
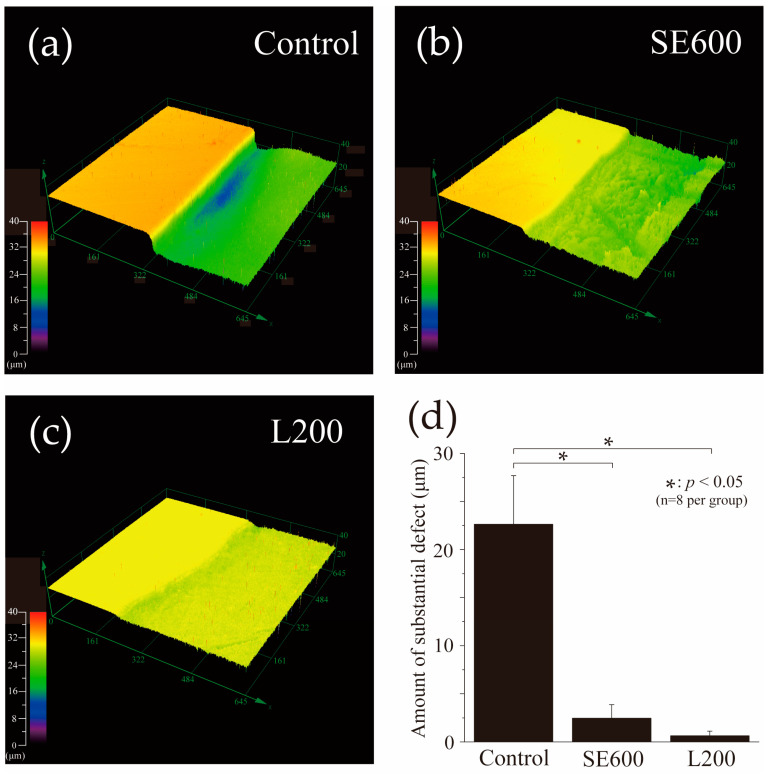
Step height profiles were measured using a 3D laser microscope. Boundary images of RS and ES of (**a**) control, (**b**) SE600, and (**c**) L200 groups after acid challenge. The left sides of figure (**a**–**c**) show the RS protected by wax and not demineralized. The right side shows an ES exposed to acid for a long period. (**d**) Graphical representation of substantial defects due to demineralization (*n* = 8/group, * *p* < 0.05).

Figure 3 shows 3D laser microscopy measurements of enamel surface profiles after pH cycling. The left side of Figure 3a–c depicts the RS, which was not demineralized and was protected with wax, whereas the right side shows the ES. The control group exhibited the largest mean defect, with amount of 22.63 ± 5.07 μm. Significant differences were observed among all other groups (*p* < 0.05). The SE600 group demonstrated a reduced defect of 2.46 ± 1.41 μm, approximately one-tenth of that in the control group, whereas the L200 group displayed a further reduction to 0.64 ± 0.48 μm. However, no significant difference was observed between the two groups (*p* > 0.05).

### 3.3. Polarizing Microscopy Images of Enamel Cross-Sections

**Figure 4:**
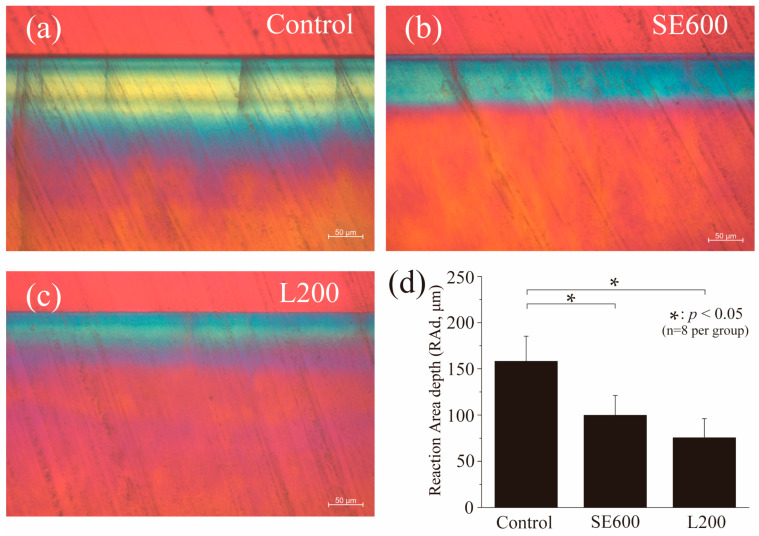
Polarizing microscopy images of enamel cross-sections after pH cycling. Cross-sectional images of the experimental surface after the acid challenge were obtained by polarizing microscopy in the (**a**) control, (**b**) SE600, and (**c**) L200 groups. Scale bar: 50 μm. (**d**) Graphical representation of the reaction area depth due to demineralization (*n* = 8, * *p* < 0.05).

Figure 4 presents the graphs and RAd images obtained from a polarizing microscope after the acid challenge. Yellow and blue polarized regions in the control group are consistently visible from the enamel surface to its deep layer, with an RAd of 158.14 ± 27.26 μm (Figure 4a). The RAd in the SE600 group decreased to 99.75 ± 21.47 μm, with an increase in the blue polarized region (Figure 4b). The RAd in the SE600 group was significantly smaller than that in the control group (*p* < 0.05, Figure 4d). The RAd in the L200 group was lower than that in both the control and SE600 groups (75.36 ± 20.63 μm) (Figure 4c,d), although no significant difference was found between the SE600 and L200 groups (*p* > 0.05, Figure 4d).

### 3.4. Cross-Sectional SEM Observations After pH Cycling

**Figure 5:**
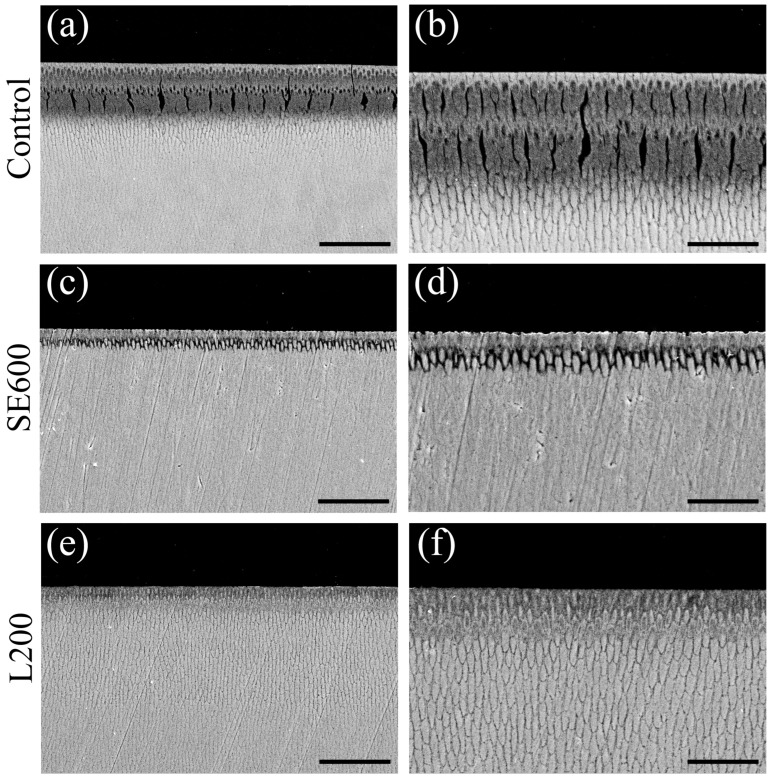
SEM images of enamel cross-sections after pH cycling. Cross-sectional SEM images of control (**a**,**b**), SE600 (**c**,**d**), and L200 (**e**,**f**) groups. (**a**,**c**,**e**) Scale bar: 50 μm (500-fold magnification). (**b**,**d**,**f**) Scale bar: 25 μm (1000-fold magnification). (**a**–**f**) Images were recorded after carbon evaporation from the sample.

Figure 5 shows secondary electron images of the vertically cut samples after pH cycling. The control group displayed an acid-resistant layer with a higher signal intensity than the surrounding tissue, which was located approximately 5 μm from the surface. Severe superficial demineralization occurred 25 to 50 μm below the surface, with enlarged enamel rod gaps and collapsed rod structures (Figure 5a,b). Demineralization images displayed multiple layers, indicating that the enamel was subjected to repeated demineralization and remineralization cycles. The SE600 group depicted a smaller area of decreased signal intensity than the control group owing to demineralization (Figure 5c). Nonetheless, the enamel prism structure collapsed, with widened prism spaces observed just below the approximately 5 μm thick acid-resistant layer (Figure 5d). A gradient-like signal intensity decrease was observed from the surface in the L200 group, with mild enamel rod structure destruction within 5 to 10 μm of the surface (Figure 5e,f). The enamel structure deeper than 20 to 30 μm from the surface in the L200 group remained, similar to that of the healthy enamel.

### 3.5. Quantitative Analysis of Mineral Loss by Microradiography

**Figure 6:**
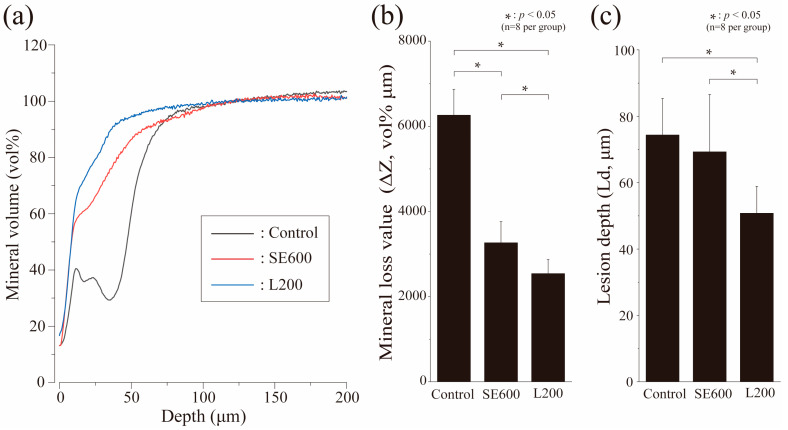
Graphical representation of ΔZ and Ld after pH cycling. (**a**) Mineral values by tooth depth: control (black), SE600 (red), and L200 (blue). (**b**) ΔZ (*n* = 8, * *p* < 0.05): mean ± SD. (**c**) Ld (*n* = 8, * *p* < 0.05): the demineralization depth was determined to be up to 95% of that of healthy enamel.

Figure 6 presents a graph depicting the changes in the mineral content (vol%) with depth using microradiography, including mineral loss (ΔZ, vol%μm) and demineralization depth (Ld, μm) for each group. The control group exhibited a bimodal graph with a high intensity at 15 and 25 μm from the surface and a notable decrease in the intensity between 20 and 40 μm due to demineralization (Figure 6a). This graph demonstrates a typical mineral loss profile of subsurface enamel demineralization. Although the mineral content recovery in the SE600 group began at a shallow depth of 0 to 10 μm, the slope moderated from 60 vol%, with a gradual recovery up to 100 μm (Figure 6a). The L200 group displayed a similar shallow-depth recovery at 0 to 10 μm, surpassing 90 vol% by 40 to 50 μm (Figure 6a).

The ΔZ for the control group was 6260.75 ± 609.28 vol%μm, which was significantly higher than that of all other groups (*p* < 0.05, Figure 6b). The SE600 group exhibited reduced mineral loss, approximately half that of the control group, (3266.38 ± 491.92 vol%μm. The L200 group exhibited the smallest value at 2543.05 ± 331.33 vol%μm, which was significantly different from that of the SE600 group (*p* < 0.05, Figure 6b). The Ld of the control group was 74.36 ± 10.97 μm, the largest among all groups. The Ld of the SE600 group was 69.30 ± 17.26 μm, a decrease compared with the control group, although insignificant (*p* > 0.05, Figure 6c). The L200 group had the smallest Ld at 50.81 ± 8.09 μm, significantly different from that of all other groups (*p* < 0.05, Figure 6c).

### 3.6. Development of an IFRD for Human Maxilla Using 3D Layering Technology

**Figure 7:**
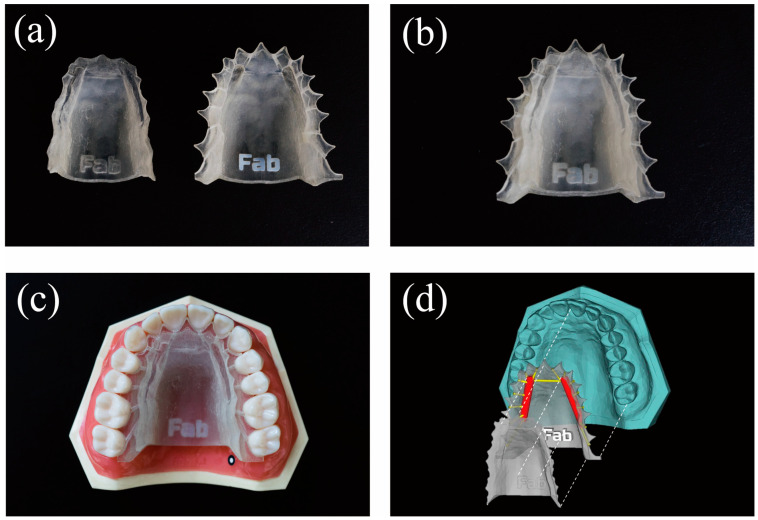
IFRD design for the human maxilla using 3D layering technology. (**a**) Upper (left) and lower (right) parts of the IFRD tray. (**b**) Assembled trays with integrated parts via mechanical interlocking. (**c**) Tray positioned on the maxillary jaw model (diagram of oral cavity use). (**d**) Schematic showing the assembly of each part and the maxillary teeth jaw model. Red represents the gel reservoir tank for the IFRD, and yellow indicates the flow pipe.

A prototype IFRD for the human maxilla was created using optical impressions and 3D layering technology (Figure 7). The tray consisted of two mechanically interlocked parts: upper and lower (Figure 7a,b). A combination of the grooves inside both parts formed a reservoir tank. A 0.5 mm diameter hollow flow pipe extended from the tank to the cervical area of each tooth. The saliva entering the tray was directed to the tank via capillary action, releasing F^−^ into the saliva. As the gel in the tank diluted and disintegrated with saliva, F^−^ was delivered to the cervical area through the flow pipe. The reservoir tank inside the tray stored approximately 200 mg of fluoride-releasing gel. The prototype tray was fitted to the maxillary tooth model, and mechanical retention was confirmed using an undercut area design (Figure 7c).

## 4. Discussion

The fluoride release experiment demonstrated that the F^−^ concentration was approximately five to eight times higher in the L200 group than in the SE600 group (Figure 2). Both SE600 and L200 gels were derived from the same hydroxy cellulose and differed only in the presence of cationization. Free F^−^ ions in the solution and saliva were monovalent anions with a negative charge [1,2,3,4]. We hypothesized that the positively charged L200 group would adsorb more F^−^ than the SE600 group through van der Waals forces due to cationization [23,24]. The experimental results supported this hypothesis, indicating that the L200 group adsorbed more F^−^ and released a substantial amount of it into the saliva upon dissolution in water. This in vitro experiment involved a reaction between the gel and mechanically stirred distilled water at 37 °C, which did not completely replicate the conditions in the human oral cavity. Specifically, the dissolution rate of the gel in the oral cavity was expected to be slower because of the slow saliva secretion rate and clearance interval [34]. Previous studies have demonstrated that cationized HEC has lower water solubility than non-cationized HEC [23,24]. The F^−^ concentration in the L200 group continued to increase gradually, even after 120 min (Figure 2), likely because of the lower solubility and longer dissolution time of cationized HEC compared to the SE600 group. The IFRD gel for IFRD in this study satisfied the following five requirements: (1) it disintegrated with water and artificial saliva, (2) it had a high viscosity and remained stable in a flowing environment, (3) it adsorbed F^−^ through cationization, (4) it offered sustained fluoride release, and (5) it maintained a neutral pH upon dissolution. The L200 group fulfilled these requirements and is currently considered the optimal gel for IFRD. Cationic HEC, a proven safe toothpaste ingredient, has been extensively studied [23,24,25]. A company-developed toothpaste with cationic HEC demonstrated a 1.6-fold improvement in F^−^ retention in teeth [35]. An experiment mimicking oral care for initial enamel demineralization using cationized HEC toothpaste twice daily for 1 week indicated a 30% improvement in remineralization [35,36]. These findings highlighted the efficacy of cationized HEC in preventing caries and enhancing fluoride retention in the oral cavity.

We next analyzed the 3D laser microscopy measurements of surface profiles and polarizing microscopy images of enamel cross-sections after pH cycling (Figure 3 and Figure 4). The SE600 and L200 groups demonstrated significantly suppressed demineralization and reduced enamel defects and RAd compared with the control group, indicating their effectiveness in enhancing enamel acid resistance and reducing defects. The SEM histological analysis revealed superficial demineralization with trabecular collapse in the control group, whereas the SE600 and L200 groups depicted improved trabecular structures and reduced demineralization (Figure 5). The changes in the refractive index and narrowed intertrabecular spaces in SEM images were consistent with those reported previously on improved enamel acid resistance [31,37,38]. Both qualitative and quantitative analyses, including microradiography (Figure 6), demonstrated that compared with the SE600 group, the L200 group exhibited significant reductions in ΔZ and Ld. These findings suggest that the use of cationic gels enhances enamel acid resistance.

Several previous studies have investigated the relationship between salivary F^−^ concentration and demineralization inhibition [39,40,41]. In vitro research reported that demineralization was inhibited at fluoride concentrations of 0.02–0.1 ppm in artificial saliva and significantly inhibited at 0.1–0.5 ppm [1,4,7,40]. Similarly, salivary fluoride concentrations of 0.1–0.5 ppm inhibited demineralization and enhanced remineralization in vivo [1,40]. In particular, the presence of 1.0 ppm F^−^ in the saliva decreases the critical pH of the enamel from 5.5 to 4.9, demonstrating a strong inhibitory effect on demineralization and promotion of remineralization [1,4,8]. The concentration of salivary fluoride was 0.017 to 0.026 ppm in non-fluoridated areas, whereas it was only 0.032 to 0.047 ppm in fluoridated areas, not reaching 0.1 ppm [39,41]. Thus, resting salivary fluoride levels are inadequate to actively inhibit demineralization or promote remineralization [39,41]. Although glass bead-type IFRDs supply fluoride at 0.2 to 0.5 ppm, potentially effective in preventing caries, it releases less than 1.0 ppm F^−^, which is insufficient for varying individual salivary secretions and habits [12,13,19,20]. The SE600 group released 0.682 ± 0.079 mg/L fluoride and the L200 group released 4.241 ± 0.825 mg/L fluoride at 60 min, far exceeding the conventional IFRD concentrations (Figure 2). The use of SE600 and L200 gels significantly elevated salivary fluoride levels and enhanced acid resistance and remineralization.

Compared to high-concentration topical fluoride applications, an IFRD provides a sustained release of low F^−^ concentrations in the saliva over extended periods [12,19]. High-concentration topical fluoride, such as that from dental clinics or toothpaste, temporarily elevates F^−^ levels in the mouth; however, these levels rapidly diminish after application [3,6]. Continuous F^−^ release is more effective than intermittent F^−^ release in preventing caries [1,2,3,4,6,8]. Recent studies demonstrated that long-term application of low-concentration fluoride with loosely bonded fluoride (loosely bonded F) is more critical in inhibiting demineralization than the formation of fluorapatite with high-concentration fluoride [1,12,19,42]. Loosely bonded F, adsorbed on enamel, exists as “CaF_2_-like” spherical particles in neutral or alkaline pH and releases F^−^ under acidic conditions, increasing Ca^2+^ and F^−^ saturation and inhibiting demineralization [6,42,43,44,45]. In vitro studies have demonstrated that 1 ppm fluoride or higher is necessary for the formation of loosely bonded F [1,4,8,41]. The L200 group consistently supplied low fluoride concentrations to artificial saliva, significantly forming loosely bonded F on enamel surfaces and strongly suppressing demineralization. Additionally, the slowly released F^−^ in the oral cavity is expected to diffuse into the saliva and penetrate the plaque, increasing the F^−^ concentration [4,8].

Numerous studies, including randomized controlled trials (RCTs) and animal experiments, have examined the caries-preventive effects of IFRDs [12,19,20]. An RCT involving 63 children (31 in the intervention and 32 in the control groups) found a significantly lower caries rate in the intervention group (DMFT: mean −0.72, 95% confidence interval [CI]: −1.23 to −0.21; DMFS: mean −1.52, 95% CI: −2.68 to −0.36) [9,19]. Mirth et al. reported that rats with IFRDs (0.15 mg/day sustained-release F dose) and distilled water had significantly fewer caries and lower caries scores than those in the placebo group [13]. Singh et al. reviewed different fluoride-releasing devices and suggested their potential for high-risk groups but emphasized the need for further clinical trials for widespread use [12,19]. The limited use of IFRDs is attributed to the high technical skills required and costs involved for their construction and design, the need for dentist maintenance, discomfort, and the inability to self-apply [10,12,13,19]. We applied 3D printing technology to design an IFRD to simultaneously mold multiple designs, including complex structures, at a low cost [27]. This innovation allows patients to attach and remove devices themselves, thereby improving their comfort and maintenance compared to conventional IFRDs (Figure 7) [26,27,46]. The tray-type IFRD was designed for self-use by the patient, in which the reservoir was filled with the gel and placed in the mouth. The gel disintegrates upon contact with saliva for approximately half a day, releasing F^−^ (Figure 2). The release rate can be controlled by adjusting the viscosity of the gel, allowing customization based on individual risk levels. This device is highly effective for use before bedtime, when the risk of caries is elevated, as it regulates the duration and quantity of fluoride release. It is applicable to general caries prevention, industrial hygiene, and hospital ward management, where oral care is challenging. The gel can be applied at the start of a shift, and the tray can be cleaned and reused post-shift. Its flexibility allows it to fit inside dentures and orthodontic appliances and is beneficial for patients undergoing intermaxillary fixation post-jaw surgery or in settings such as nursing homes where frequent oral care is difficult. Additionally, hyposalivation and mucosal diseases can be treated by modifying gel ingredients. In addition to caries prevention, IFRDs will advance preventive dental care personalized for each patient’s oral condition and symptoms.

Because the study was conducted in an in vitro artificial oral environment, it has limitations. In addition, further experiments are needed to determine the chemical properties and physical properties of the gel, as well as the mechanism of its reaction with tooth structures. The preparation method of our gel follows published patents and references and is identified in the patents as “hydroxyethylcellulose dimethyldiallylammonium chloride,” a type of cellulose. The only difference between the gel production method in the patent and this study is the dehydration time in the rotary evaporator. The mixing of HEC with sodium fluoride and the dialysis treatment performed in this study did not result in any chemical changes, and it is believed that the physical properties of the raw material are the same as those of cellulose, except for the adsorption of fluoride ions. Being a model experiment, the plaque was not present immediately after brushing, making it difficult to ascertain the amount of fluoride penetrating the plaque. However, previous studies have reported that the amount of fluoride in plaque increases when an IFRD is worn and that it binds to calcium and phosphate inside the plaque, affecting the enamel surface and plaque bacteria. A similar effect is expected with the IFRD [1,3,12,19]. Further experiments are required to determine its behavior in the oral cavity.

## 5. Conclusions

We developed a gel for IFRD using histological and engineering techniques, which can release effective fluoride concentrations to inhibit demineralization. The fluoride release rate of the gel increased from 0.682 ± 0.079 mg/L to 4.241 ± 0.825 mg/L upon cationization. The mineral loss of L200 group exhibited the smallest value at 2543.05 ± 331.33 vol%μm, which was significantly different from that of the SE600 group (*p* < 0.05). These results suggested that cationization enhances caries-inhibitory effects on enamel. In addition, we designed and printed a new palatially mounted IFRD that could be loaded with a gel tailored to the patient’s oral cavity using optical impressions and 3D printing technology, confirming that the gel was stored and maintained on a model. Altogether, this device facilitates self-care application and offers a new option for high-risk patients and postoperative management, where oral care is challenging.

## Data Availability

All data have been included in the manuscript.

## Supplementary Materials

The following supporting information can be downloaded at: https://www.mdpi.com/article/10.3390/ma17235731/s1, Figure S1: Properties of two types of gels for IFRD created in this study.
