# Supplementary material for: Improving Enamel Acid Resistance by an Intraoral Fluoride-Release Device Incorporating Cationic Hydroxy Cellulose Gel Using 3D Printer Molding
Source: Materials (Basel). 2024 Nov 23;17(23):5731. doi: 10.3390/ma17235731 (PMC11642292; doi:10.3390/ma17235731)
Supplement: Supplementary file 1 [file materials-17-05731-s001.zip › materials-3277466-supplementary.pdf]

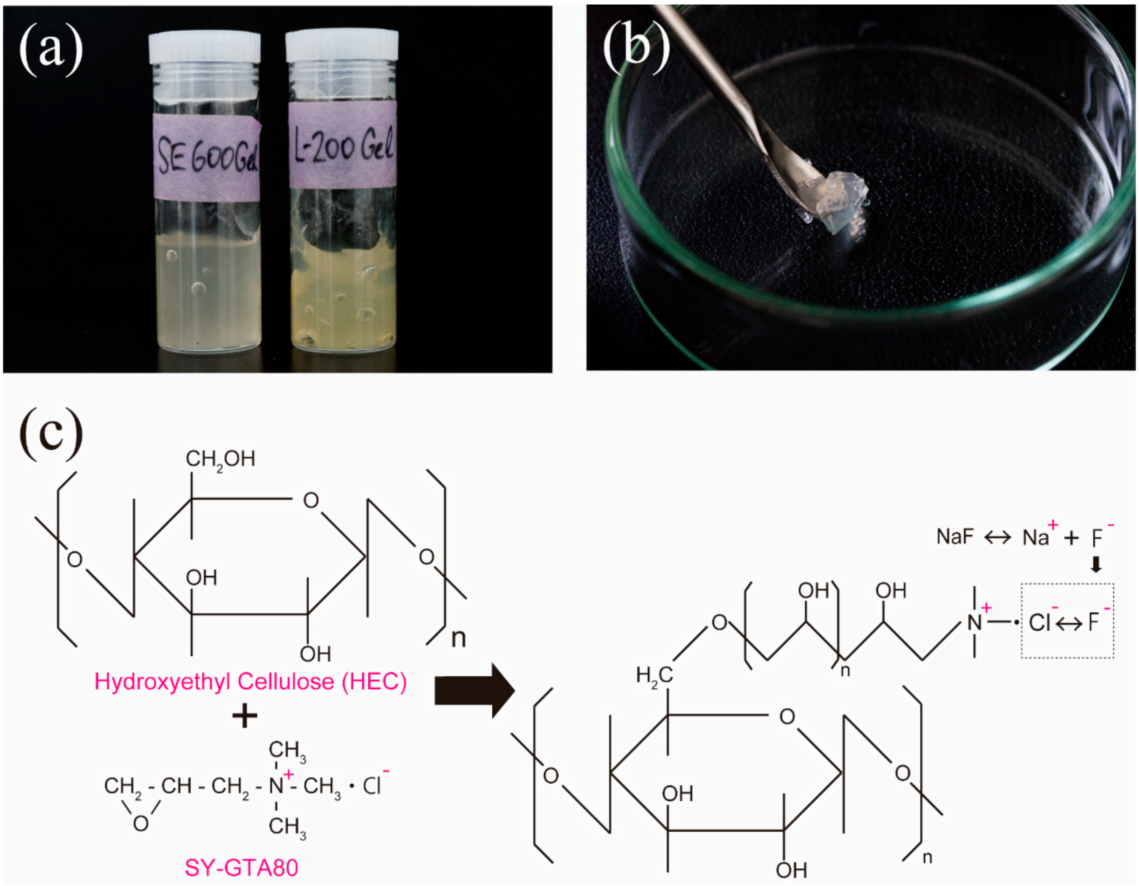

Supplementary Figure S1. Properties of two types of gels for IFRD created in this study.

(a) Images of HEC gel (SE600) and cationized HEC gel (L200) stored in ampules. (b) Image of L200 placed on a Petri dish showing elastic non-fluid gel-like properties. (c) Manufacturing method of L-200 and a hypothetical theory for introducing F ions by ion replacement in an aqueous solution are demonstrated.
